# Supplementary material for: Water binding and hygroscopicity in π-conjugated polyelectrolytes
Source: Nat Commun. 2023 Jul 5;14:3978. doi: 10.1038/s41467-023-39215-9 (PMC10322933; doi:10.1038/s41467-023-39215-9)
Supplement: Supplementary file 1 — Supplementary Information [file 41467_2023_39215_MOESM1_ESM.pdf]

Supplementary Information

Water binding and hygroscopicity in π-conjugated polyelectrolytes

Cindy Guanyu Tang,[1] Mazlan Nur Syafiqah,[2] Qi-Mian Koh,[2] Mervin Chun-Yi Ang,[2] Kim-Kian Choo,[2] Ming-Ming Sun,[2] Martin Callsen,[1] Yuan-Ping Feng,[1] Lay-Lay Chua,[2]* Rui-Qi Png,[1]* Peter K.H. Ho[1]*

[1] Department of Physics, National University of Singapore, Lower Kent Ridge Road, S117550, Singapore
[2] Department of Chemistry, National University of Singapore, Lower Kent Ridge Road, S117552, Singapore

* Correspondence to:
L.L.C. (chmcll@nus.edu.sg), R.Q.P. (ruiqi@nus.edu.sg) or P.K.H.H. (phyhop@nus.edu.sg)

Contents

1. Supplementary Figures

Supplementary Figure 1.	AMBER molecular mechanics simulation of mTFF-SO_3-Na	2
Supplementary Figure 2.	Water binding motifs in $(Na^+ X^-)_r (H_2O)_p$	3
Supplementary Figure 3.	Water binding motifs in $(Li^+ X^-)_r (H_2O)_p$ and $(K^+ X^-)_r (H_2O)_p$	4
Supplementary Figure 4.	Water binding motifs in $(TMA^+ X^-)_r (H_2O)_p$	5

2. Supplementary Tables

Supplementary Table 1.	Chemical structure and water desorption data of polyelectrolytes	6
Supplementary Table 2.	Kinetics *A* and E_a parameters for type-II and type-III water and binding motif assignments	14
Supplementary Table 3.	Validation of OPLS4 for water binding	16
Supplementary Table 4.	Water binding energies and *ν*OH frequencies for hydrated ion multiplets	17

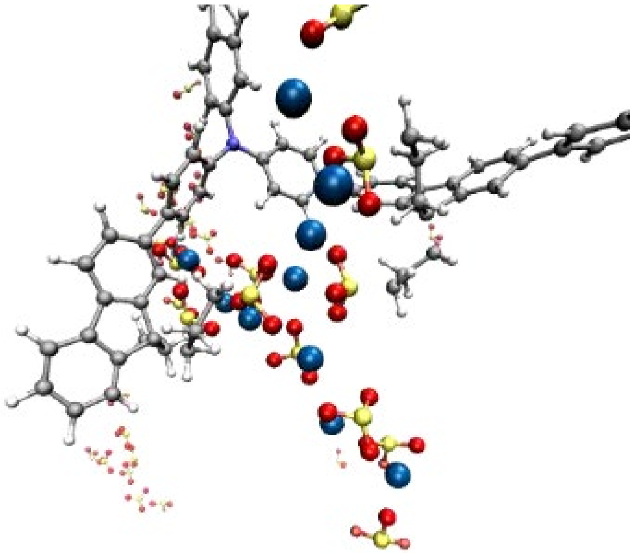

Supplementary Figure 1. AMBER molecular mechanics simulation of mTFF-SO_3-Na. The computed morphology comprises a nanoscale segregation of ions in clusters from the polymer matrix: blue atoms, Na; red, O; yellow, S; grey, C; white, H; purple, N, rendered in perspective mode, with alkyl chains omitted for clarity. The chemical structure of this polymer is given in S/N 3, Supplementary Table 1.

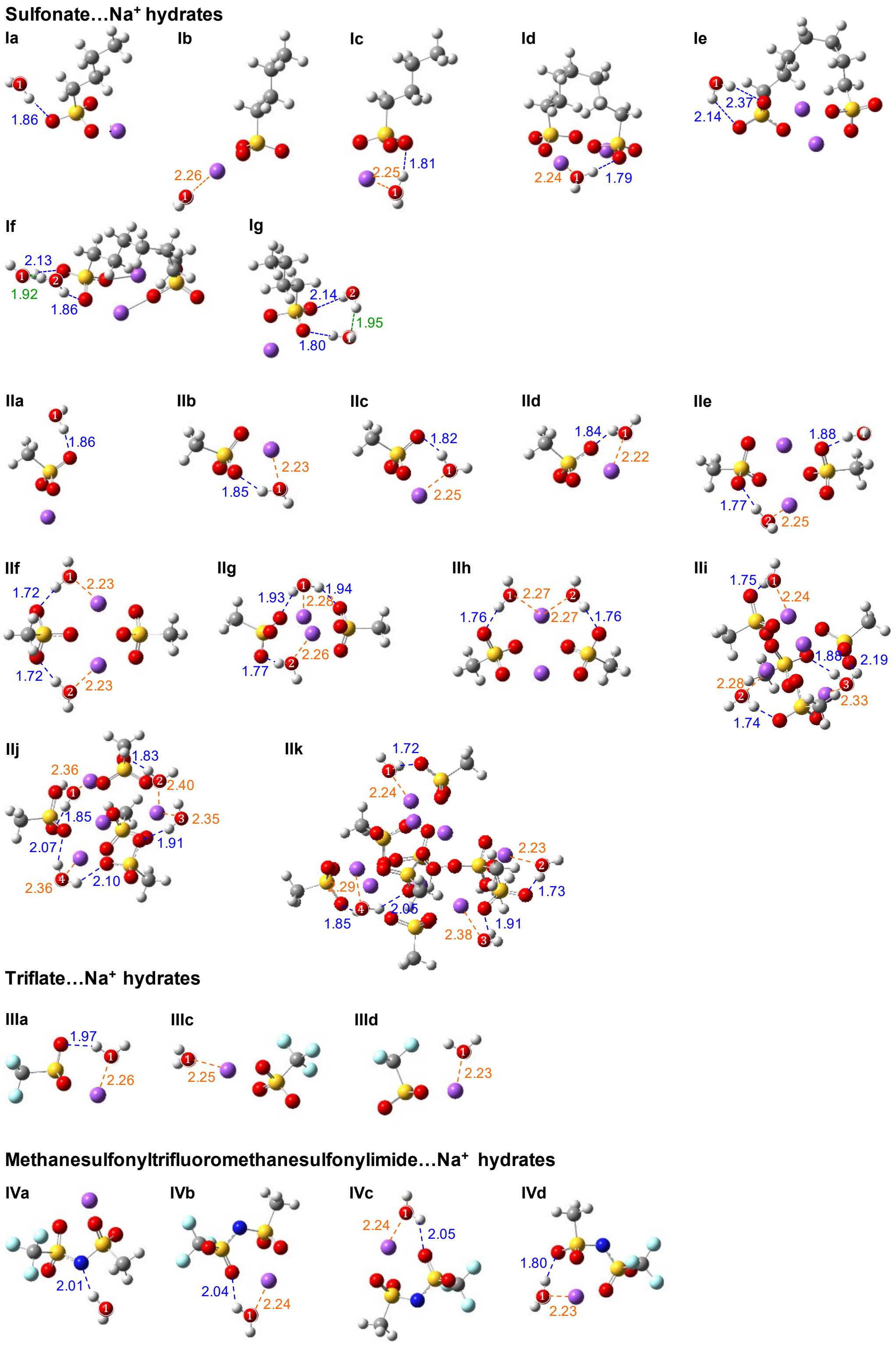

Supplementary Figure 2. Water binding motifs in $(Na^+ X^-)_r$ $(H_2O)_p$. Methodology: DFT/CAM-B3LYP/6-31++G(*d*,*p*). Bonded distance given in Å from water to: anion (blue dashed line), cation (orange) and H-bonded water (green), with water molecules numbered. Atom legend: violet, Na; red, O; yellow, S; cyan, F; grey, C; white, H.

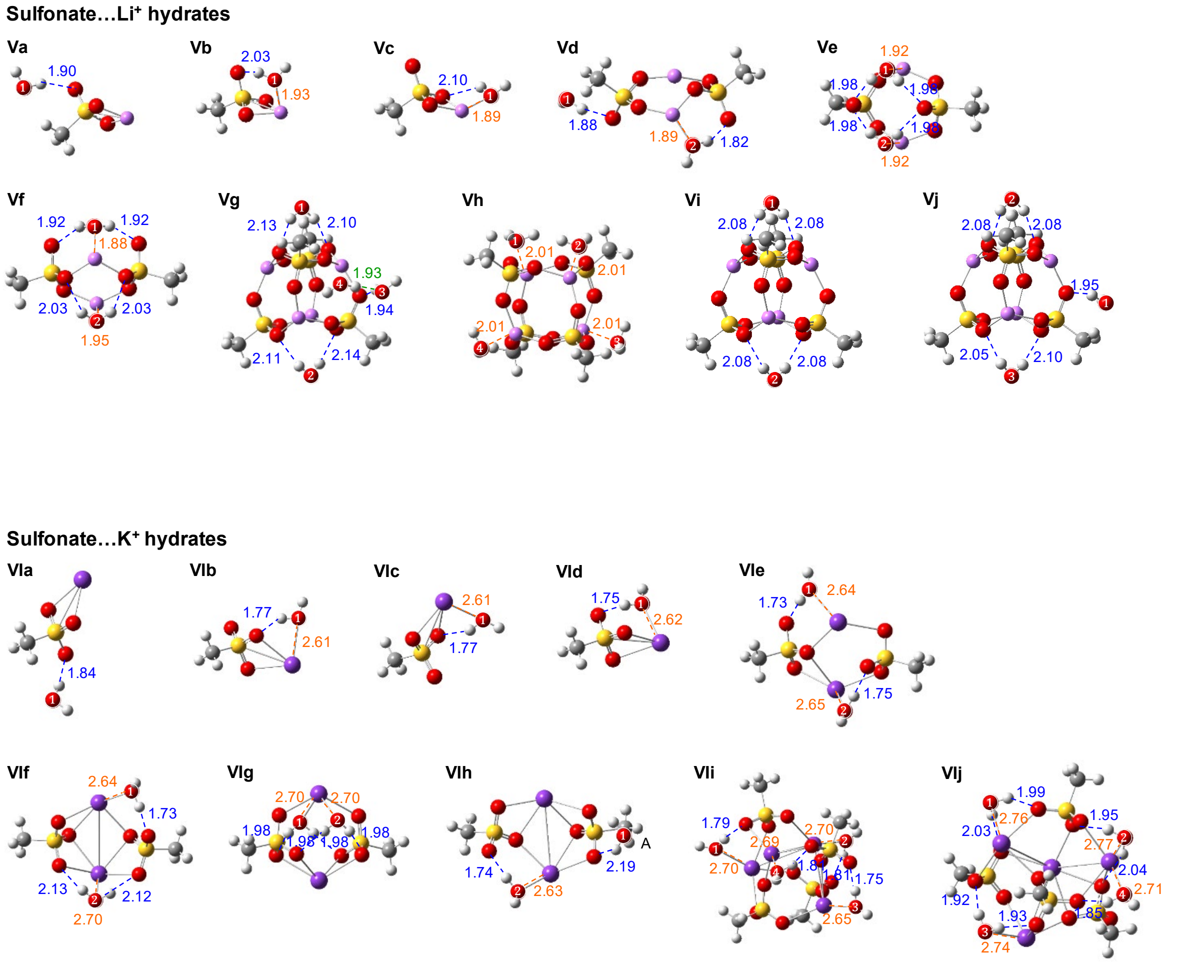

Supplementary Figure 3. Water binding motifs in $(Li^+ X^-)_r$ $(H_2O)_p$ and $(K^+ X^-)_r$ $(H_2O)_p$. Methodology: DFT/CAM-B3LYP/6-31++G(*d*,*p*). Bonded distance given in Å from water to: anion (blue dashed line), cation (orange) and H-bonded water (green), with water molecules numbered. Atom legend: violet, Li or K; red, O; yellow, S; cyan, F; grey, C; white, H.

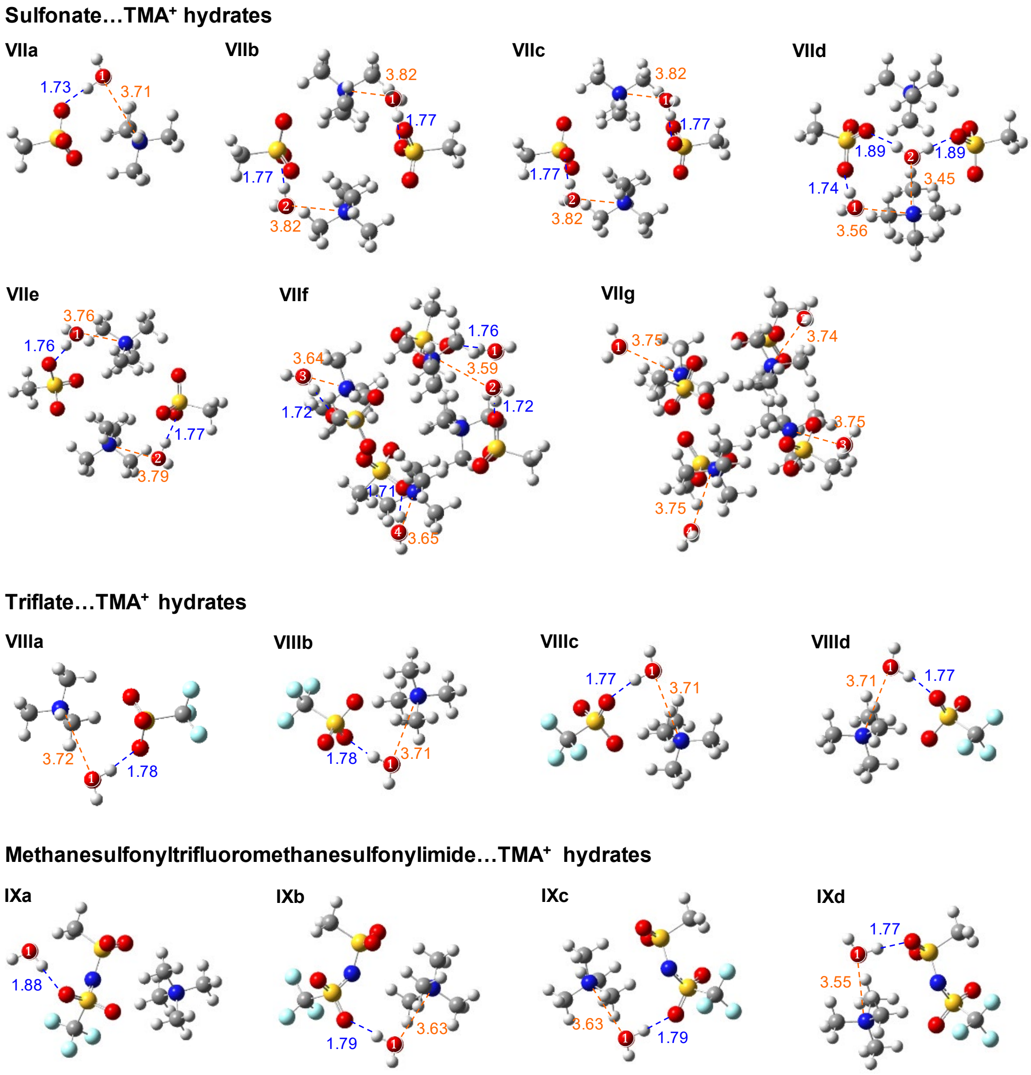

Supplementary Figure 4. Water binding motifs in (TMA^+ X^-)$_r$ (H_2O)$_p$. Methodology: DFT/CAM-B3LYP/6-31++G(*d*,*p*). Bonded distance given in Å from water to: anion (blue dashed line), cation (orange) and H-bonded water (green), with water molecules numbered. Atom legend: red, O; yellow, S; blue, N; cyan, F; grey, C; white, H.

Supplementary Table 1. Chemical structure and water desorption data of polyelectrolytes

S/N	Material	Ion pair		Scan number	Hydration number (mol H_2O/mol ion pair)[a]				Sample preparation[d]
		Fixed ion	Free ion		Type-I (QCM)[b]	Type-I (TGA)[c]	Type-II (TGA)[c]	Type-III (TGA)[c]	
**Non-conjugated polyelectrolytes**									
1	PSSNa<br>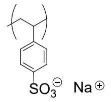	$–SO_3^–$	Na^+	1st	2.2* 4.9† 7.3‡	0.83	75°C (45–105°C)<br>0.70	not present	as-received sample; QCM film spun from H_2O soln
				2nd	--	0.88	80°C (50–110°C)<br>0.68	not present	
2	PVB-NMe3-Cl<br>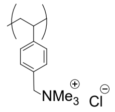	$–NMe_3^+$	$Cl^–$	1st	--	0.41	75°C (40–160°C)<br>1.56	--	pptd by $CHCl_3$ from MeOH soln; sample degrades beyond 165°C
				2nd	--	0.40	80°C (40–160°C)<br>1.57	--	
**Conjugated polyelectrolytes: Tethered sulfonate and perfluoroalkylsulfonylimidosulfonyl polyelectrolytes**									
3	mTFF-SO_3-Na<br>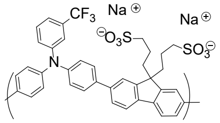	$–SO_3^–$	Na^+	1st	2.7* 5.2† 7.3‡	1.22	80°C (45–135°C)<br>0.98	220°C (180°C–)<br>0.57	ppted by H_2O from DMSO soln; QCM film spun from MeOH soln
				2nd	--	0.88	80°C (45–135°C)<br>0.97	220°C (180°C–)<br><0.02	
4	*p*-doped mTFF-SO_3-Na<br>DL = 0.77 h^+/r.u.	$–SO_3^–$	Na^+	1st	2.6* 4.1† 5.2‡	nd	nd	nd	sample prepd in SC form by contact-doping after spinning undoped film from MeOH soln, and baking at 150°C, 15min
				2nd	2.0* 3.6† 4.7‡	--	--	--	

5	TFB-SO_3-Na<br>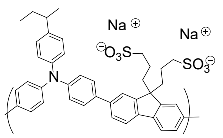	$-SO_3^-$	Na^+	1st	--	1.19	70°C (45–100°C)<br>0.92	210°C (170°C–)<br>0.66	pptd by H_2O from DMSO soln
				2nd	--	0.81	80°C (50–110°C)<br>0.93	210°C (170°C–)<br><0.02	
6	TFB-SO_3-Na<br>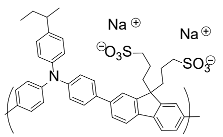	$-SO_3^-$	Na^+	1st	--	1.10	85°C (45–130°C)<br>0.95	220°C (190°C–)<br>0.12	ppted by $CHCl_3$ from DMSO soln
				2nd	--	0.67	90°C (45–130°C)<br>0.85	220°C (190°C–)<br><0.02	
7	TFB-SO_3-TMA<br>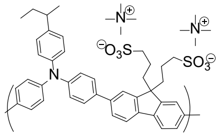	$-SO_3^-$	NMe_4^+	1st	--	1.69	60°C (40–80°C)<br>0.93	200°C (150°C–)<br>1.83	pptd by H_2O from DMSO soln
				2nd	--	1.31	65°C (40–90°C)<br>0.67	200°C (150°C–)<br><0.02	
8	TFB-SO_3-TEA<br>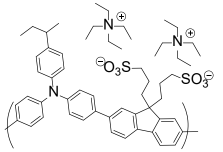	$-SO_3^-$	NEt_4^+	1st	--	1.75	60°C (40–80°C)<br>1.00	190°C (145°C–)<br>1.72	pptd by H_2O from DMSO soln
				2nd	--	1.18	65°C (40–90°C)<br>0.75	190°C (145°C–)<br><0.02	
9	TFB-CF_3SIS-Li<br>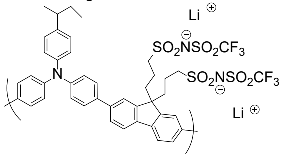	$-SO_2N^-$<br>SO_2CF_3	Li^+	1st	2.7* 4.8† 7.2‡	1.12	70°C (50–130°C)<br>0.99	215°C (190°C–)<br>0.10	pptd by DEE from ACN soln; QCM film spun from ACN soln
				2nd	--	1.10	70°C (50–130°C)<br>0.93	215°C (190°C–)<br><0.02	
10	TFB-CF_3SIS-Na<br>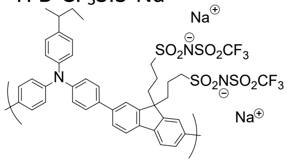	$-SO_2N^-$<br>SO_2CF_3	Na^+	1st	1.3* 3.0† 4.6‡	0.56	60°C (40–110°C)<br>0.65	210°C (170°C–)<br>0.13	pptd by DEE from ACN soln; QCM film spun from ACN soln
				2nd	--	0.33	60°C (40–110°C)<br>0.26	210°C (170°C–)<br><0.02	

11	TFB-CF_3SIS-Cs 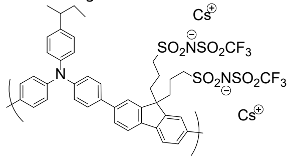	$–SO_2N^–$ SO_2CF_3	Cs^+	1st	1.5* 2.4† 4.1‡	0.43	50°C (40–60°C)<br>0.08	210°C (170°C–)<br>0.06	pptd by DEE from ACN soln; QCM film spun from ACN soln
				2nd	--	0.23	50°C (40–60°C)<br>0.04	210°C (170°C–)<br><0.02	
12	TFB-CF_3SIS-TMA 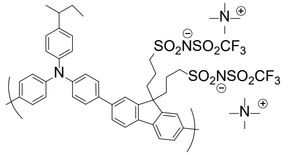	$–SO_2N^–$ SO_2CF_3	NMe_4^+	1st	0.8* 1.2† 1.6‡	0.35	50°C (40–70°C)<br>0.11	210°C (170°C–)<br>0.09	pptd by DEE from ACN soln; QCM film spun from ACN soln
				2nd	--	0.27	55°C (40–70°C)<br>0.05	215°C (170°C–)<br><0.02	
13	TFB-CF_3SIS-TEA 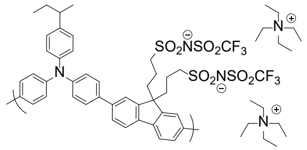	$–SO_2N^–$ SO_2CF_3	NEt_4^+	1st	--	0.24	120°C (50-150°C)<br>0.08	210°C (170°C–)<br>0.09	pptd by DEE from ACN soln
				2nd	--	0.25	50°C (40–70°C)<br>0.05	210°C (170°C–)<br><0.02	
14	TFB-CF_3SIS-TPP 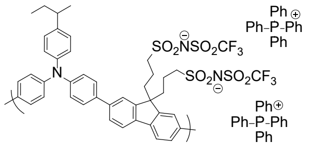	$–SO_2N^–$ SO_2CF_3	PPh_4^+	1st	--	0.25	100°C (80-120°C)<br>0.47	--	pptd by DEE from ACN soln; sample degrades beyond 220°C
				2nd	--	<0.02	100°C (80-120°C)<br>0.04	--	
15	TFB-C_2F_5SIS-Na 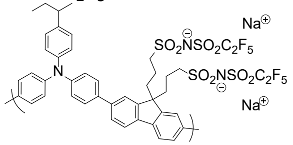	$–SO_2N^–$ $SO_2C_2F_5$	Na^+	1st	--	0.33	70°C (50–100°C)<br>0.46	210°C (180°C–)<br>0.08	pptd by DEE from ACN soln
				2nd	--	0.33	70°C (50–100°C)<br>0.26	210°C (180°C–)<br><0.02	
16	mTFF-CF_3SIS-Na 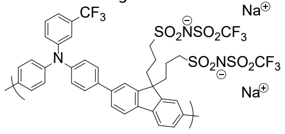	$–SO_2N^–$ SO_2CF_3	Na^+	1st	2.1* 4.2† 5.9‡	0.78	70°C (50–100°C)<br>0.45	220°C (180°C–)<br>0.11	pptd by DEE from ACN soln; QCM film spun from ACN soln
				2nd	--	0.35	70°C (50–100°C)<br>0.31	220°C (180°C–)<br><0.02	

17	mTFF-CF_3SIS-TMA<br>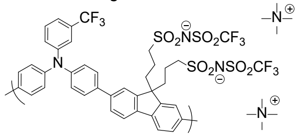	–SO_2N^-<br>SO_2CF_3	NMe_4^+	1st	--	0.36	50°C (40–70°C)<br>0.10	190°C (145–230°C)<br>0.14	pptd by DEE from ACN soln
				2nd	--	0.27	55°C (45–75°C)<br>0.03	190°C (145–230°C)<br><0.02	
18	mTFF-C_2F_5SIS-Na<br>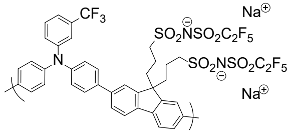	–SO_2N^-<br>$SO_2C_2F_5$	Na^+	1st	2.7* 4.8† 7.2‡	1.13	60°C (40–90°C)<br>0.49	210°C (170°C–)<br>0.05	pptd by DEE from ACN soln; QCM film spun from ACN soln
				2nd	--	1.05	60°C (40–90°C)<br>0.39	210°C (170°C–)<br><0.02	
19	*p*-doped mTFF-C_2F_5SIS-Na<br>DL = 0.77 h^+/r.u.	–SO_2N^-<br>$SO_2C_2F_5$	Na^+	1st	1.4* 2.4† 4.0‡	0.87	100°C (50–150°C)<br>1.31	--	sample prepd in SC form by soln-doping; QCM film spun from ACN soln; sample de-dopes after 1st scan
				2nd	--	1.05	60°C (40–90°C)<br>0.13	--	
20	pTFF-C_2F_5SIS-Na<br>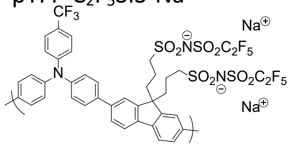	–SO_2N^-<br>$SO_2C_2F_5$	Na^+	1st	2.3* 4.5† 6.5‡	nd	nd	nd	pptd by DEE from ACN soln; QCM film spun from ACN soln
21	*p*-doped pTFF-C_2F_5SIS-Na<br>DL = 0.87 h^+/r.u.	–SO_2N^-<br>$SO_2C_2F_5$	Na^+	1st	2.7* 3.7† 4.2‡	nd	nd	nd	sample prepd in SC form by soln-doping; QCM film spun from ACN soln

Conjugated polyelectrolytes: Tethered trimethylammonium polyelectrolytes

22	F8-NMe3-TfO<br>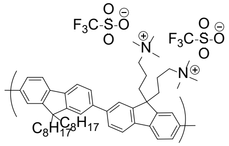	$-NMe_3^+$	TfO^-	1st	--	0.64	130°C (90–180°C)<br>3.35	210°C (180°C–)<br>0.30	pptd by DEE from DMF soln
				2nd	--	0.24	130°C (90–180°C)<br><0.02	210°C (180°C–)<br><0.02	
23	F8N-NMe3-TfO<br>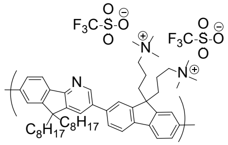	$-NMe_3^+$	TfO^-	1st	--	0.53	120°C (80–160°C)<br>1.78	200°C (160°C–)<br>0.07	pptd by DEE from DMF soln
				2nd	--	0.77	120°C (80–160°C)<br><0.02	200°C (160°C–)<br><0.02	
24	TFB-NMe3-TfO<br>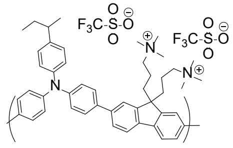	$-NMe_3^+$	TfO^-	1st	1.4* 2.1† 2.5‡	0.63	115°C (80–150°C)<br>1.08	210°C (150°C–)<br>0.17	pptd by DEE from DMF soln; QCM film spun from MeOH soln
				2nd	--	0.50	115°C (80–150°C)<br><0.02	210°C (150°C–)<br><0.02	
25	FBT-NMe3-TfO<br>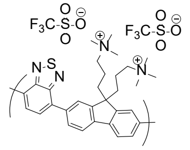	$-NMe_3^+$	TfO^-	1st	--	0.62	110°C (75–125°C)<br>0.22	210°C (160°C–)<br>0.50	pptd by DEE from DMF soln
				2nd	--	0.42	110°C (75–125°C)<br><0.02	210°C (160°C–)<br><0.02	
26	NDITT-NMe3-TfO<br>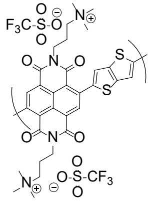	$-NMe_3^+$	TfO^-	1st	--	0.50	70°C (40–100°C)<br>0.19	170°C (115–225°C)<br>0.88	pptd by DEE from DMF soln
				2nd	--	0.53	70°C (40–100°C)<br>0.16	170°C (115–225°C)<br><0.02	

27	*n*-doped NDITT-NMe3-TfO<br>DL = 1.0 e^-/r.u.	$–NMe_3^+$	$TfO^–$	1st	--	1.57	100°C (40–170°C)<br>2.23	200°C (170°C –)<br>1.51	sample prepd in SC form by soln-doping in DMSO, then pptd by diglyme; sample dedopes after 1st scan
				2nd	--	0.22	<0.02	<0.02	
28	NDITT-NMe3-I<br>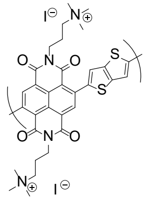	$–NMe_3^+$	$I^–$	1st	--	1.17	60°C (40–80°C)<br>0.50	--	pptd by DEE from DMF soln; sample degrades beyond 160°C
				2nd	--	nd	nd		
29	NDITT-NMe3-TFSI<br>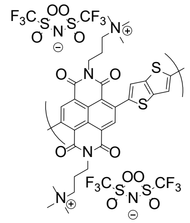	$–NMe_3^+$	$TFSI^–$	1st	--	0.13	140°C (95–190°C)<br>1.40	220°C (200°C –)<br>0.30	pptd by DEE from DMF soln; sample degrades beyond 230°C
				2nd	--	<0.02	140°C (95–190°C)<br><0.02	220°C (200°C –)<br><0.02	
30	NDITT-NMe3-BArF<br>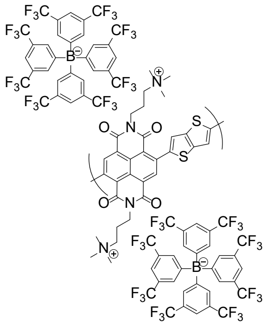	$–NMe_3^+$	$BArF^–$	1st	--	0.18	140°C (110–175°C)<br>6.42	225°C (200°C –)<br>1.96	pptd by DEE from DMF soln
				2nd	--	<0.02	140°C (110–175°C)<br><0.02	225°C (200°C –)<br><0.02	

Charge-doped π-conjugated polymers (non-polyelectrolytes)

31	*p*-doped TFB:SbF_6<br>DL = 0.6 h^+/r.u.<br>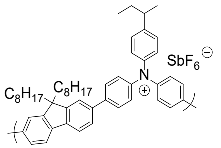	--	--	1st	0.8* 1.2† 1.4‡	0.64	--	--	sample prepd in doped form in $CHCl_3$:ACN (10:3), then pptd by DMC; QCM film spun from $CHCl_3$:ACN soln; SbF_6^- decomposes above 150°C
32	*n*-doped PNDI:$CoCp_2$<br>DL = 1.0 e^-/r.u.	--	--	1st	--	0.46	--	--	sample prepd in doped form in THF:DMSO (8:1); then dried under vacuum without exposing to ambient; $CoCp_2^+$ decomposes at 130°C

Footnotes:

[a] Water hydration number, given in mole ratio of water molecules to ion formula units (equivalently, ion pairs) for different types of water sorbed in the polymer. nd = not determined

[b] QCM methodology. 30-nm-thick films were spin-cast in air, or nitrogen glove-box (doped materials), onto QCM quartz crystal, and mounted into flow cell. Dry N_2 (<10 ppm H_2O) was flowed through cell at 295 K until quartz resonance frequency stabilized. Wet nitrogen at relative humidity (RH) of:
*58% (bubbling through saturated NaBr solution), followed by
†85% (saturated KCl solution), and then
‡97.5% (saturated K_2SO_4),
was flowed into cell, after stabilization of film mass at each RH. The hydration number is computed from frequency change, assuming density of polymer film is 1.45 g cm^{-3}. Only type-I water can be observed this way. The hydration number is found to be independent of film thickness between 12 nm to 1.5 µm.

[c] Different sorbed water species, classified according to desorption characteristics: Type-I water, desorption in dry N_2 below 50°C; type-II water, desorption between 50 and 150°C; type-III water, desorption above 150°C. The hydration numbers are computed from weight loss data, based on the dry weight of the polymer remaining at the highest temperature. For polymers that degrade before this temperature, the dry weight corresponds to that just before decomposition. The temperature at the maximum loss rate is given to indicate the characteristic temperature of the step. The extrapolated onset and end temperatures are given inside brackets. Where only the onset temperature is given, the extrapolated end temperature lies above the ceiling temperature in the ramp segment. Type-I water is significantly under-reported due to desorption before the initial isothermal segment is reached.

[d] **Preparation method for powder samples for TG.** Solvents: methanol (MeOH), dimethyl sulfoxide (DMSO), acetonitrile (ACN), diethyl ether (DEE), *N*,*N*-dimethylformamide (DMF), dimethyl carbonate (DMC), chloroform ($CHCl_3$), tetrahydrofuran (THF). The precipitated materials were dried in vacuum oven at overnight (80°C, 10^{-2} mbar), equilibrated in the nitrogen glovebox for at least 16 h, and then loaded into thermo-analyzer in ambient (22°C, 65% RH). Dopant: *p*-dopant is $NOSbF_6$ in ACN, *n*-dopant is decamethylcobaltocene ($CoCp_2$) in DMSO. DL is doping level, determined by UV-Vis; GB, nitrogen glovebox; SC, self-compensated; pptd, precipitated; soln, solution; prepd, prepared. **Preparation method for films for QCM.** Powder samples, either in their undoped or doped form, were dissolved in their respective solvents for spin-casting. Undoped films were baked at 120°C for 10 min prior to measurement.

Supplementary Table 2. Kinetics *A* and E_a parameters for type-II and type-III water, and binding motif assignments

S/N	Material	Type-II	Type-III	Fitted range/ comment[a]	Binding motif assignment[b]
**Tethered sulfonate polyelectrolytes**					
1	mTFF-SO_3-Na	A = 1.1 s^{-1}<br>E_a = 0.18 eV	A = 6.6 x 10^4 s^{-1}<br>E_a = 0.72 eV	II: 62–117°C<br>III: 152–243°C<br>pptd with water	II: α<br>III: δ, caged
		A = nd<br>E_a = nd	A = 3.5 x 10^7 s^{-1}<br>E_a = 0.96 eV	different sample:<br>III: 166–238°C	II: nd<br>III: δ, caged
2	TFB-SO_3-Na	A = 0.36 s^{-1}<br>E_a = 0.15 eV	A = 36 s^{-1}<br>E_a = 0.40 eV	II: 69–103°C<br>III: 163–225°C<br>pptd with water	II: α<br>III: δ
3	TFB-SO_3-TMA	A = 1.8 s^{-1}<br>E_a = 0.19 eV	A = 4.8 s^{-1}<br>E_a = 0.32 eV	II: 59–81°C<br>III: 136–216°C<br>pptd with water	II: α<br>III: δ
4	TFB-SO_3-TEA	A = 1.4 s^{-1}<br>E_a = 0.18 eV	A = 6.9 s^{-1}<br>E_a = 0.34 eV	II: 63–90°C<br>III: 115–227°C<br>pptd with water	II: α<br>III: δ
5	PSSNa	A = 0.34 s^{-1}<br>E_a = 0.15 eV	A = --<br>E_a = --	II: 60–113°C<br>III: negligible	II: α
**Tethered trifluoromethylsulfonylimidosulfonyl polyelectrolytes**					
6	TFB-CF_3SIS-Li	A = 1.2 s^{-1}<br>E_a = 0.20 eV	A = nd<br>E_a = nd	II: 64–149°C<br>III: nd	II: α<br>III: nd
7	TFB-CF_3SIS-Na	A = 2.0 s^{-1}<br>E_a = 0.20 eV	A = 46 s^{-1}<br>E_a = 0.44 eV	II: 56–115°C<br>III: 195–223°C	II: α<br>III: δ
8	TFB-CF_3SIS-Cs	A = 4.1 x 10^2 s^{-1}<br>E_a = 0.31 eV	A = nd<br>E_a = nd	II: 48–65°C<br>III: nd	II: α<br>III: nd
9	TFB-CF_3SIS-TMA	A = 1.7 x 10^2 s^{-1}<br>E_a = 0.28 eV	A = 3.5 s^{-1}<br>E_a = 0.33 eV	II: 45–57°C<br>III: 136–217°C	II: α<br>III: δ
10	TFB-CF_3SIS-TPP	A = 1.9 x 10^6 s^{-1}<br>E_a = 0.64 eV	A = --<br>E_a = --	II: 62–120°C<br>III: negligible	II: δ, caged
**Tethered trimethylammonium polyelectrolytes**					
11	F8-NMe3-TfO	A = 1.4 x 10^2 s^{-1}	A = 1.1 x 10^4 s^{-1}	II: 56–151°C	II: δ

		E_a = 0.40 eV	E_a = 0.62 eV	III: 200–214°C	III: δ, caged
12	F8N-NMe3-TfO	A = 10 s^{-1}<br>E_a = 0.29 eV	A = --<br>E_a = --	II: 90–170°C<br>III: negligible	II: δ
13	TFB-NMe3-TfO	A = 82 s^{-1}<br>E_a = 0.35 eV	A = nd<br>E_a = nd	II: 95–121°C<br>III: nd	II: δ
14	FBT-NMe3-TfO	A = 18 s^{-1}<br>E_a = 0.31 eV	A = 164 s^{-1}<br>E_a = 0.46 eV	II: 96–139°C<br>III: 166–222°C	II: δ<br>III: δ, caged
15	NDITT-NMe3-TfO	A = 11 s^{-1}<br>E_a = 0.21 eV	A = 9.3 s^{-1}<br>E_a = 0.33 eV	II: 60–106°C<br>III: 108–204°C	II: α<br>III: δ
16	NDITT-NMe3-I	A = 3.4 x10^3 s^{-1}<br>E_a = 0.39 eV	A = --<br>E_a = --	II: 49–94°C<br>III: dec above 160°C	II: δ
17	NDITT-NMe3-TFSI	A = 12 s^{-1}<br>E_a = 0.32 eV	A = nd<br>E_a = nd	II: 105–187°C<br>III: nd	II: δ
18	NDITT-NMe3-BArF	A = 1.8 x10^4 s^{-1}<br>E_a = 0.56 eV	A = 2.6 x 10^4 s^{-1}<br>E_a = 0.70 eV	II: 75–177°C<br>III: 194–228°C	II: δ, caged<br>III: δ, caged
19	PVB-NMe3-Cl	(a) A = 2.9 s^{-1}<br>E_a = 0.26 eV<br>(b) A = 0.42 s^{-1}<br>E_a = 0.16 eV	A = --<br>E_a = --	II:<br>(a) 53–80°C<br>(b) 97–142°C<br>III: decd above 165°C	II(a): α<br>(diffusion<br>limited?)<br>II(b): α

Footnotes:

[a] Temperature range for activation energy analysis of type-II and type-III water. Fitting correlation coefficient R^2 is better than 0.99 over indicated temperature range, which typically spans at least 75% of the temperature interval between extrapolated onset and end. Accuracy of kinetic parameters is limited in some cases by the available temperature range. Overlapping steps are split at inflection point. nd = not determined, pptd = precipitated, decd = decomposed

[b] Binding motifs: α = anion; β = bridging, i.e. two anions; χ = cation; δ = dual, i.e. cation–anion pair; caged = water molecule nestled between ions inside cluster

Supplementary Table 3. Validation of OPLS4 for water binding

Computed quantity	OPLS4	PM3	DFT/CAM-B3LYP/6-31++G(*d,p*)	Reference
**Water dimer [H_2O…HOH]:**				
O...O distance	2.74 Å	3.03 Å	2.85 Å	2.97 Å[a]
dimer binding energy[b]	0.30 eV	0.08 eV	0.20 eV	0.14 eV[a]
**Hydrated sodium sulfonate ion pair [$CH_3SO_3^-$ Na^+ (H_2O)]:**				
α binding[b]	0.48 eV	0.33 eV	0.30 eV	--
δ binding[b]	1.02 eV	0.59 eV	0.81 eV	--

Footnotes:

[a] Experimental data obtained from: [1] J.A. Odutola and T.R. Dyke, *J. Chem. Phys.* 72 (1980) 5062; and [2] B.E. Rocher-Casterline, L.C. Ch'ng, A.K. Mollner and H. Reisler. *J. Chem. Phys.* 134 (2011) 211101

[b] OPLS4 overestimates binding energy in water dimer, due to over-shortening of the hydrogen bond length. OPLS4 also overestimates binding energies to $CH_3SO_3^-$ Na^+ by 0.2 eV for both α binding (i.e. H-bond to sulfonate) and δ binding (i.e. H-bond to sulfonate and electrostatic bond to Na^+). PM3 underestimates δ binding energy, due to overestimation of Na^+ radius (computed Na…O distance: PM3, 3.12 Å; OPLS4, 2.34 Å; DFT, 2.25 Å).

Supplementary Table 4. Water binding energies and *v*OH frequencies for hydrated ion multiplets

Structure[a]	Binding motif[b]	ΔU_o (eV)[c]	ν_LOH (cm^{-1})[d]	ν_UOH (cm^{-1})[d]
**Na^+ RSO_3^-:**				
**I**a	α	0.30	3530 [471]	3729 [92]
**I**b	χ	0.63	3667 [45]	3740 [140]
**I**c	δ	0.81	3349 [864]	3735 [106]
**I**d	δ	0.79	3390 [758]	3734 [122]
**I**e	β	0.27	3633 [83]	3697 [71]
**I**f	α (+H_2O)<br>α (+H_2O)		①: 3527 [400]<br>②: 3569 [395]	①: 3731 [129]<br>②: 3663 [195]
**I**g	α (+H_2O)<br>α (+H_2O)		①: 3473 [616]<br>②: 3569 [276]	①: 3724 [89]<br>②: 3660 [191]
**II**a	α	0.30	3531 [466]	3730 [92]
**II**b	δ	0.78	3453 [556]	3740 [125]
**II**c	δ	0.80	3357 [768]	3736 [104]
**II**d	δ	0.74	3474 [520]	3743 [138]
**II**e	α<br>δ	0.51	①: 3553 [406]<br>②: 3407 [768]	①: 3729 [53]<br>①: 3729 [165]
**II**f	δ<br>δ	0.70	①+②: 3366 [591]<br>①+②: 3350 [1262]	①+②: 3736 [32]<br>①+②: 3735 [198]
**II**g	δ+α<br>δ	0.68	①: 3546 [219]<br>②: 3416 [743]	①: 3582 [513]<br>②: 3726 [108]
**II**h	δ<br>δ	0.68	①+②: 3410 [1288]<br>①+②: 3402 [316]	①+②: 3736 [204]<br>①+②: 3735 [1]
**II**i	δ<br>δ<br>δ+α	0.59	①: 3394 [807]<br>②: 3351 [842]<br>③: 3536 [391]	①: 3740 [117]<br>②: 3727 [95]<br>③: 3674 [158]
**II**j	δ<br>δ<br>δ<br>δ+α	0.51	①: 3451 [498]<br>②: 3469 [501]<br>③: 3546 [351]<br>④: 3600 [105]	①: 3723 [97]<br>②: 3724 [106]<br>③: 3698 [121]<br>④: 3649 [260]
**II**k	δ<br>δ<br>δ<br>δ+α	0.56	①: 3354 [730]<br>②: 3362 [894]<br>③: 3563 [293]<br>④: 3523 [293]	①: 3734 [119]<br>②: 3741 [115]<br>③: 3729 [93]<br>④: 3637 [390]

**Na^+ $CF_3SO_3^-$:**				
**III**a	δ	0.68	3487 [422]	3735 [130]
**III**c	χ	0.68	3665 [47]	3737 [146]
**III**d	χ	0.70	3644 [104]	3742 [190]
**Na^+ $(CH_3SO_2)(CF_3SO_2)N^-$:**				
**IV**a	α (imide **N**)	0.30	3569 [353]	3730 [121]
**IV**b	δ (CF_3**SO_2**)	0.68	3607 [163]	3744 [174]
**IV**c	δ (CF_3**SO_2**)	0.68	3607 [163]	3744 [175]
**IV**d	δ (CH_3**SO_2**)	0.75	3432 [684]	3733 [131]
**Li^+ RSO_3^-:**				
**V**a	α	0.28	3561 [357]	3732 [106]
**V**b	δ	0.76	3477 [346]	3719 [117]
**V**c	δ	0.84	3580 [146]	3736 [156]
**V**d	α<br>δ	0.61	①: 3546 [427]<br>②: 3460 [550]	①: 3731 [103]<br>②: 3727 [146]
**V**e	δ+α<br>δ+α	0.91	①+②: 3541 [17]<br>①+②: 3536 [297]	①+②: 3600 [890]<br>①+②: 3582 [0.1]
**V**f	δ+α<br>δ+α	0.83	①: 3525 [158]<br>②: 3574 [145]	①: 3578 [497]<br>②: 3615 [417]
**V**g	β<br>β<br>α (+H_2O)<br>type-I	0.25	①: 3630 [135]<br>②: 3633 [114]<br>③: 3552 [344]<br>④: 3575 [262]	①: 3685 [144]<br>②: 3690 [121]<br>③: 3693 [163]<br>④: 3723 [146]
**V**h	χ<br>χ<br>χ<br>χ	0.43	①+②: 3634 [7]<br>①+②: 3634 [66]<br>③+④: 3632 [22]<br>③+④: 3632 [48]	①+②: 3709 [345]<br>①+②: 3707 [0.73]<br>③+④: 3707 [136]<br>③+④: 3707 [72]
**V**i	β<br>β	0.26	①+②: 3629 [218]<br>①+②: 3630 [71]	①+②: 3681 [41]<br>①+②: 3681 [298]
**V**j	α<br>β<br>β	0.25	①: 3527 [273]<br>②+③: 3630 [204]<br>②+③: 3631 [90]	①: 3729 [111]<br>②+③: 3682 [111]<br>②+③: 3684 [231]
**K^+ RSO_3^-:**				
**VI**a	α	0.32	3509 [560]	3728 [83]

**VI**b	δ	0.71	3383 [832]	3736 [101]
**VI**c	δ	0.71	3384 [831]	3736 [101]
**VI**d	δ	0.78	3309 [1011]	3735 [90]
**VI**e	δ<br>δ	0.56	①+②: 3358 [873]<br>②+①: 3387 [933]	①+②: 3731 [96]<br>①+②: 3737 [93]
**VI**f	δ<br>δ+α	0.52	①: 3387 [908]<br>②: 3600 [90]	①: 3732 [103]<br>②: 3675 [281]
**VI**g	δ+α<br>δ+α	0.60	①+②: 3560 [209]<br>①+②: 3556 [134]	①+②: 3616 [982]<br>①+②: 3608 [0.2]
**VI**h	α<br>δ	0.43	①: 3632 [89]<br>②: 3373 [936]	①: 3688 [66]<br>②: 3730 [99]
**VI**i	δ<br>δ<br>δ<br>δ	0.54	①: 3444 [717]<br>②: 3402 [587]<br>③: 3412 [928]<br>④: 3493 [563]	①: 3734 [115]<br>②: 3734 [94]<br>③: 3735 [86]<br>④: 3738 [118]
**VI**j	δ+α<br>δ+α<br>δ+α<br>δ	0.46	①: 3585 [170]<br>②: 3570 [240]<br>③: 3547 [311]<br>④: 3550 [408]	①: 3648 [396]<br>②: 3631 [347]<br>③: 3625 [318]<br>④: 3739 [145]
**TMA^+ $MeSO_3^-$:**				
**VII**a	δ	0.58	3352 [982]	3728 [72]
**VII**b	δ<br>δ	0.44	①+②: 3423 [582]<br>①+②: 3424 [944]	①+②: 3726 [104]<br>①+②: 3726 [49]
**VII**c	δ<br>δ	0.44	①+②: 3423 [579]<br>①+②: 3424 [947]	①+②: 3726 [112]<br>①+②: 3726 [41]
**VII**d	δ<br>δ+α	0.42	①: 3393 [853]<br>②: 3522 [245]	①: 3727 [76]<br>②: 3584 [751]
**VII**e	δ<br>δ	0.44	①+②: 3417 [570]<br>②+①: 3421 [1026]	①+②: 3728 [78]<br>①+②: 3727 [80]
**VII**f	α<br>δ<br>δ<br>δ	0.38	①: 3429 [762]<br>②: 3370 [962]<br>③: 3399 [985]<br>④: 3384 [968]	①: 3728 [91]<br>②: 3722 [78]<br>③: 3728 [71]<br>④: 3725 [81]
**VII**g	χ<br>χ<br>χ<br>χ	0.11	①+②+③+④:<br>3664 [21]<br>3664 [23]<br>3664 [15]<br>3664 [17]	①+②+③+④:<br>3746 [61]<br>3746 [67]<br>3746 [118]<br>3746 [60]
**TMA^+ $CF_3SO_3^-$:**				
**VIII**a	δ	0.52	3433 [766]	3726 [90]

**VIII**b	δ	0.52	3437 [780]	3726 [91]
**VIII**c	δ	0.52	3437 [779]	3726 [91]
**VIII**d	δ	0.52	3436 [780]	3726 [91]
**TMA$^+$ (CH_3SO_2)(CF_3SO_2)N$^-$**				
**IX**a	α (CF_3**SO_2**)	0.26	3560 [429]	3728 [110]
**IX**b	δ (CF_3**SO_2**)	0.52	3485 [745]	3724 [113]
**IX**c	δ (CF_3**SO_2**)	0.52	3486 [744]	3724 [113]
**IX**d	δ (CH_3**SO_2**)	0.57	3411 [895]	3724 [96]
**References**				
**H_2O**			3667 [8]	3754 [66]
**$(H_2O)_2$**	HOH…OH_2	0.20	donor: 3578 [378]<br>acceptor: 3666 [17]	donor: 3731 [99]<br>acceptor: 3748 [99]

Footnotes:

[a] Structure labels correspond to those of Figure 5 and Supplementary Figures 2–4. Computed by DFT with CAM-B3LYP/6-31++G(*d*,*p*).

[b] Binding motif labels: α, H-bonded to anion; β, H-bond bridging two anions; χ, electrostatic bond to cation; δ, dual bonded to cation and anion; δ+α, combination bond to cation and two anions; (+H_2O) indicates additional H-bonding to water molecule; boldface atoms in brackets denote binding group. The number of binding motifs reported corresponds to the value of *p* in $(M^+ X^-)_r (H_2O)_p$.

[c] ΔU_o is the average adiabatic binding energy, zero-point-energy corrected, for all water molecules in the structure at 0 K.

[d] ν_LOH and ν_UOH give the scaled frequencies of the lower and upper νOH bands, respectively, where scaled frequency is obtained from DFT frequency ν' according to: $\nu = \nu' (1.184 - 0.00006 \nu')$. Values in square brackets give the relative infrared absorption cross sections. Numbers in circles are water molecule labels corresponding to those in Figure 5 and Supplementary Figures 2–4. Addition sign denotes combination band.
