# Supplementary material for: Water binding and hygroscopicity in π-conjugated polyelectrolytes
Source: Nat Commun. 2023 Jul 5;14:3978. doi: 10.1038/s41467-023-39215-9 (PMC10322933; doi:10.1038/s41467-023-39215-9)
Supplement: Supplementary file 3 — Description of Additional Supplementary files [file 41467_2023_39215_MOESM3_ESM.pdf]

Description of Additional Supplementary Items

File name: Supplementary Video 1

Description: 360°-view of 8 hydrated ion clusters, total length 2 min 30 s

Gaaiho PDF Trial
www.gaaiho.com
